# Frequency and Clinical Epidemiology of Canine Monocytic Ehrlichiosis in Dogs Infested with Ticks from Sinaloa, Mexico

**DOI:** 10.1155/2013/797019

**Published:** 2013-10-24

**Authors:** Carolina Guadalupe Sosa-Gutierrez, Maria Teresa Quintero Martinez, Soila Maribel Gaxiola Camacho, Silvia Cota Guajardo, Maria D. Esteve-Gassent, María-Guadalupe Gordillo-Pérez

**Affiliations:** ^1^Unidad de Investigacion Medica en Enfermedades Infecciosas y Parasitarias, Hospital de Pediatria, Centro Medico Nacional Siglo XXI, Instituto Mexicano del Seguro Social, 07300 Mexico City, Mexico; ^2^Departamento de Parasitologia, Facultad de Medicina Veterinaria y Zootecnia, Universidad Nacional Autonoma de México, Mexico City, Mexico; ^3^Departamento de Parasitologia, Facultad de Medicina Veterinaria y Zootecnia, Universidad Autonoma de Sinaloa, SIN, Mexico; ^4^Department of Veterinary Pathobiology, College of Veterinary Medicine and Biomedical Sciences, Texas A&M University, TX, USA

## Abstract

*Ehrlichia canis* is a rickettsial intracellular obligate bacterial pathogen and agent of canine monocytic ehrlichiosis. The prevalence of this disease in veterinary medicine can vary depending on the diagnostic method used and the geographic location. One hundred and fifty-two canine blood samples from six veterinary clinics and two shelters from Sinaloa State (Mexico) were analyzed in this study. All animals were suspected of having Canine Monocytic Ehrlichiosis (CME). The diagnostic methods used were the ELISA (Snap4Dx, IDEXX) together with blood smear and platelet count. From all dogs blood samples analyzed, 74.3% were positive to *E. canis* by ELISA and 40.1% were positive by blood smear. The sensitivity and specificity observed in the ELISA test were 78.8% and 86.7%. In addition, thrombocytopenia was presented in 87.6% of positive dogs. The predominant clinical manifestations observed were fever, anorexia, depression, lethargy, and petechiae. Consequently, this is the first report in which the morulae were visualized in the blood samples, and *E. canis*-specific antibodies were detected in dogs from Sinaloa, Northwest of Mexico.

## 1. Introduction

*Ehrlichia canis* is the causative agent of canine monocytic ehrlichiosis (CME). Moreover, CME is an emerging disease in veterinary medicine, and *E. canis* has been considered in the last decade as a potential zoonotic pathogen [1, 2]. It is a worldwide disease transmitted by a tick bite. The competent vector for its transmission is the Ixodidae ticks *Rhipicephalus sanguineus* and *Dermacentor variabilis* [3]. In dogs, the CME is a multiphase disorder that progresses in three stages: acute, subclinical, and chronic. Each phase is characterized by several clinical and hematologic abnormalities. Thrombocytopenia is a common finding in *E. canis* infected dogs and many clinicians tend to use it as an indication for antibiotic treatment, and it is observed in 84% of the cases and its severity varies in the different disease phases [4]. During the subclinical stage a moderate thrombocytopenia is observed, while the chronic phase is characterized by severe leukopenia and anemia. In this stage dogs show other complications such as hypocellular marrow, suppressed splenic sequestration, decreased life of platelets, and an increase of circulating migration-factor platelet inhibitor [3, 4]. The relationship between the magnitude of thrombocytopenia and prevalence of *E. canis* has been established in countries such as Brazil in 2004 where 84.1% of infected dogs showed thrombocytopenia [3]. Taken together, more data is necessary to determine the environmental factors and infected vector prevalence in Mexico due to high incidence of CME in this region of the country. The purpose of this study was to evaluate the frequency and clinical manifestations of dogs from Sinaloa, Mexico, with clinical suspicion of CME by ELISA (SNAP4Dx) and blood smears and with a history of tick infestation.

## 2. Material and Methods

### 2.1. Location

The study was conducted in Culiacan, Sinaloa, Mexico, located north 27° 02′, south 22° 29′ north latitude east 105° 23′, to 109° 28′ west longitude.

### 2.2. Samples and Serological Analysis

Samples were collected between March 2006 and July 2007, and 152 blood samples from dogs were collected in six veterinary clinics and two shelters from Sinaloa State, Mexico. Blood samples were chosen by dogs (males and females) with tick infestation and clinical signs suggesting CME (fever, anorexia, lethargy, depression, petechiae, bruising of the skin or prone to bleeding in mucous membranes, and epistaxis). Three ml of blood sample in EDTA tubes was obtained from each dog by the radial vein. All samples were processed using two techniques to detect *E. canis* specific IgG antibodies, the IDDEX ELISA kit and the Snap test. Samples were processed according to the manufacturer's recommendations. In addition, blood smears were done immediately after the blood was drawn to detect forms of *E. canis* morulae in monocytes, using Wright's stain. Finally platelet count was performed by an Automatic Hematology Analyzer (IDEXX QBC VetAutoread). All counts less than 200,000 platelets/*µ*L were considered as thrombocytopenia [4].

### 2.3. Statistical Analysis

The platelet counts from each sample were compared with the seropositive and seronegative results. Qualitative variables such as sex (female, male), age (<1 year, 1–3 years, 3–5 years, >5 years), presence of thrombocytopenia (yes, no), fever (yes, no), anorexia (yes, no), lethargy (yes, no), depression (yes, no), petechiae (yes, no), skin ecchymosis (yes, no), epistaxis (yes, no), bleeding tendency (yes, no), and anorexia (yes, no) were evaluated and used on the chi square or Fisher exact tests using EpiInfo 3.5, which provides regression analysis estimating linear 95% confidence interval (95% CI).

## 3. Results

**Figure 1:**
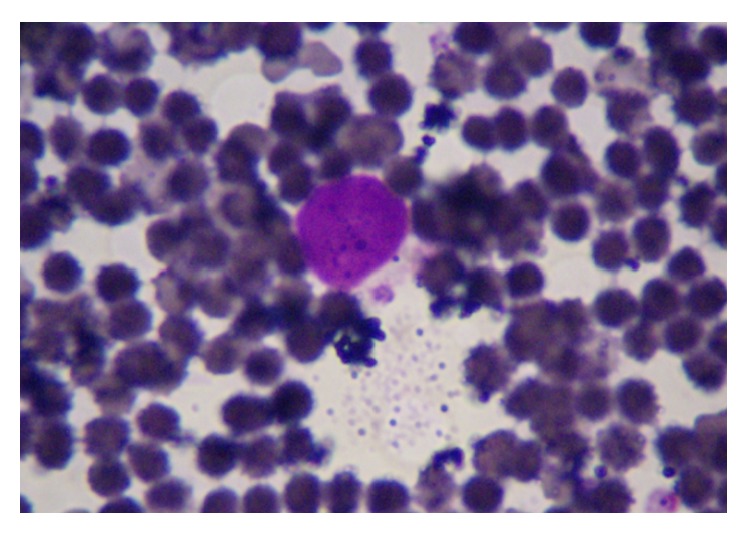
Observed using light microscopy; this is an image created from a peripheral blood smear of a dog infected with Canine Monocytic Ehrlichiosis. This image shows the morulae within the cytoplasm of a monocyte. Wright's stain.

Among the canine individuals with suspected CME 74.5% (113/152) have *E. canis*-specific antibodies. In addition, 40.1% (61/152) were found positive when examined by smear to detect *E. canis* morulae in monocytes (Figure 1). Overall the clinical manifestations observed (Table 1) were fever (91.2%), anorexia (86.7%), depression (85.0%), lethargy (72.6%), and petechiae (72.6%). The male-female ratio was 1 : 1, and the most affected age was 1-2 years old (53%), followed by under 1 year (28%) and 3–5 years (19%). We compare the results obtained from the blood smear technique and Snap 4DX; of the 152 samples analyzed; 60 (38.2%) and 21 (13.7%) were positive and negative for both methods diagnostically, with a sensibility of 78.8% (95% CI 69.8–85.6) and specificity 86.7% (95% CI 74.3–99.1), and were assessed by chi square test by calculating odds ratios (OR) and 95% confidence intervals (CI) with a 2.12 (95% CI 1.01–4.45) *P* < 0.05. The 87.6% (106) of the dogs with thrombocytopenia were positive to the presence of antibodies of *E. canis* and were evaluated by chi square test by calculating odds ratios (OD) and 95% confidence intervals (CI) with a 24.2 (95% CI 8.9–65.9) *P* < 0.01; this parameter is a risk factor for disease and showed high rates of exposure, consistent with previous reports [5].

## 4. Discussion

The results obtained in this study were similar to those observed in 2002, in which the authors compared five serodiagnostic methods for *E. canis* and found 79.2% positive samples using the same ELISA techniques as the one utilized in our study [6]. In Mexico (2000) a national study reported a 33% of seroprevalence for *E. canis* using the same diagnostic method as those presented in here [7]. Contrary to what was found in this study, our results showed the highest ehrlichiosis case. In Yucatan, Mexico, a 44.1% seroprevalence for CME has been reported using the same diagnostic method. Yucatan is in a climate zone similar to that of Sinaloa. Taken together, our study shows a frequency for CME similar if not higher than that observed in other regions of the country, as well as in other countries of Center and South America, which suggests the increasing importance of this disease in the canine population. *E. canis* has been described as a potential zoonotic pathogen for humans [5], and therefore our findings and current studies suggest its importance as human pathogen in Latin American countries.

A positive result for *E. canis* by ELISA indicates that the dog was or is exposed to this bacterial pathogen and does not necessarily imply that there is a latent infection. On the other hand, when using the ELISA test, cross-reactivity with other *Ehrlichia* species may occur since it is not specific for *E. canis*. Antibodies against this pathogen decrease approximately 6 to 9 months after infection. This together with the presence of subclinical infection in canids causes difficulties in the diagnosis of the disease by the clinicians. Consequently, there is a need of tools to support the diagnosis of the disease with hematological tests to evaluate thrombocytopenia, since this sign implies bacterial growth and its effect on antibody-producing organs.

Previous *E. canis* experimental studies [3, 4] reported thrombocytopenia in 89% of the infected dogs. In addition, thrombocytopenia was described in every stage of infection, and it persisted during subclinical stages of the disease. The morulae are easily identified by light microscopy during the acute phase of infection (4-5 days after exposure) but difficult to detect when the disease progressed undetected. Some studies reported that only 4% of the blood samples studied showed morulae in blood smears [8–10]. In this case 40.1% were found positive, which indicates that the search for the morulae in peripheral blood was done in the acute phase [11]. Moreover, dogs in endemic areas may have high IgG titers specific to *E. canis* without clinical signs and this can cause false positive serological tests [3], reinforcing the necessity for more specific test to be used in veterinary medicine.

## 5. Conclusions

In this study we have introduced the presence of the Canine Monocytic Ehrlichiosis in Sinaloa, Mexico. Dogs infested with ticks showed high frequency for *E. canis* in dogs which suggests the classification of this region of Mexico as an endemic area for the disease. Based on our findings and previous studies done in Mexico and other Latin American countries, we suggest that, in the absence of molecular methodologies for the diagnostics of CME, a serology test to detect *E. canis-*specific antibodies plus the presence of clinical manifestations and thrombocytopenia is a basic tool for its diagnosis in endemic areas.

## Figures and Tables

**Table 1:** Clinical manifestations presented by dogs positive for antibodies to *E. canis*.

Clinical manifestation	Positive *N* = 113	Frequency (%)	Odds ratio (CI)	*P* value
Fever	103	91.2	3.6 (1.3–10.2)	<0.01
Anorexia	98	86.7	4.08 (1.6–10.3)	<0.01
Lethargy	82	72.6	NS	NS
Depression	96	85.0	3.93 (1.6–9.7)	<0.01
Petechiae	82	72.6	2.5 (1.1–5.7)	<0.01
Ecchymosis	22	19.5	NS	NS
Epistaxis	80	70.8	NS	NS
Thrombocytopenia	106	93.8	5.2 (1.7–12.9)	<0.01

NS: Not Significant. Evaluated by chi square test and calculating odds ratio (OR) and 95% confidence intervals (CI).
